# Dr. Beale’s Reception

**Published:** 1856-05

**Authors:** 


*Dr. Beale's Reception.*—A reception has recently been given, by upward of fifty members of the dental profession in New York, to Dr. Beale and his family, at the house of Dr. Brown, near Jones street, to congratulate him, after the act of liberation awarded to him by the Governor of Pennsylvania. —*Boston Med. & Surg. Journal.*

